# Impact of online hemodiafiltration on bone turnover in children with CKD-5d: A prospective cohort study

**DOI:** 10.1007/s00467-025-06805-2

**Published:** 2025-05-19

**Authors:** Mohammed F. Kasem, Ragia M. Said, Aliaa Mourad, Noha R. Mohamed, Noha U. Hashem

**Affiliations:** 1 Division of Pediatric Nephrology, Department of Pediatrics, Faculty of Medicine, Ain Shams University, Cairo, Egypt; 2 Clinical Pathology Department, Faculty of Medicine, Ain Shams University, Cairo, Egypt

**Keywords:** Kidney failure, Online hemodiafiltration, Bone turnover, Chronic kidney disease-mineral bone disorder

## Abstract

**Background:**

Mineral bone disorder (MBD) is a systemic disorder associated with chronic kidney disease (CKD). Online hemodiafiltration (OL-HDF) combines hemodialysis (HD) and hemofiltration and has shown promising results in children with CKD-5d considering co-morbidities.

**Methods:**

Children with CKD-5d who were stable for at least 3 months on thrice weekly 3-h HD sessions via an arteriovenous fistula (AVF) using polysulphone membrane were shifted to post-dilution OL-HDF and followed up for 12 months. Baseline Ca, PO4, serum albumin, alkaline phosphatase, iPTH, CRP, soluble Klotho, FGF-23, BALP and TRAP-5b were assessed and repeated at the end of the 12-month follow-up period.

**Results:**

We included 31 children (17 males) with median age of 12.5 (IQR = 9.7–13.3) years and median HD vintage of 60.1 (IQR = 9.1–37.5) months. OL-HDF resulted in a statistically significant decrease in FGF-23 and FGF-23/Klotho ratios and insignificant increase in the levels of Klotho compared to their baseline values. It also led to statistically significant increase in BALP, decrease in TRAP-5b and elevation of the BALP/TRAP-5b ratio compared to their baseline values. A 12-month period of OL-HDF treatment had no significant effect on height Z-score before and after exclusion of patients having deformities of lower limbs.

**Conclusion:**

OL-HDF resulted in a significant decrease in FGF-23, TRAP-5b and FGF-23/Klotho ratio with a significant increase in BALP and BALP/TRAP ratio. This might signify a promising positive impact on bone turnover in children with CKD-5d.

**Graphical abstract:**

**Figure:**
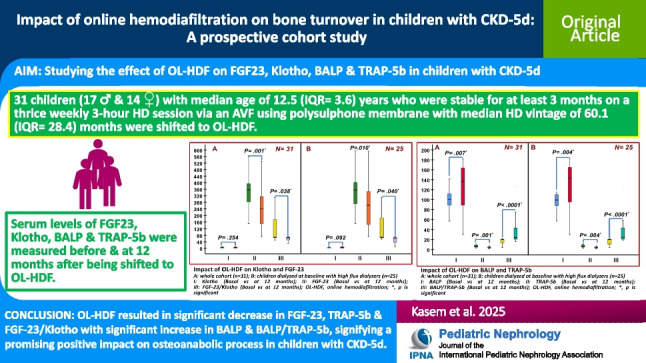
A higher resolution version of the Graphical abstract is available as A higher resolution version of the Graphical abstract is available as Supplementary information

**Supplementary Information:**

The online version contains supplementary material available at 10.1007/s00467-025-06805-2.

## Introduction

Hemodialysis (HD) clears small solutes via diffusion but is less effective in removing middle- and large-sized molecules and uremic toxins. Online hemodiafiltration (OL-HDF) is a combination of diffusion and convection in a single process that requires substitution with ultrapure fluid [1]. Research indicates that HDF can enhance survival rates when administered with a high convective dose. Better outcomes of HDF are linked to its ability to remove middle molecular weight uremic toxins including inflammatory cytokines while also promoting hemodynamic stability and reducing inflammation and oxidative stress compared to HD [2, 3].

Childhood and adolescence are critical times for building a healthy skeletal and cardiovascular system. Chronic kidney disease (CKD) disrupts mineral and bone metabolism, posing significant challenges to achieving optimal bone strength, final adult height, and cardiovascular health [4, 5]. Circulatory biomarkers, which reflect overall skeletal activity, are valuable tools for monitoring changes in bone turnover, namely, bone formation and resorption. Bone-specific alkaline phosphatase (BALP), an isoform of alkaline phosphatase (ALP) found on the surface of osteoblasts, indicates the activity of these bone-forming cells [6]. On the other hand, tartrate-resistant acid phosphatase-5b (TRAP-5b), produced by osteoclasts, serves as a marker of bone resorption as it reflects osteoclast numbers [7].

Klotho is a transmembrane protein that functions as a co-receptor of the fibroblast growth factor 23 (FGF-23) receptor and plays an essential role in maintaining phosphorus balance [8]. FGF-23 is a hormone produced by bone, playing an essential role in phosphate homeostasis by promoting its excretion by the kidney. In patients with CKD, levels of FGF-23 increase significantly, reaching peak values in those with CKD-5d. While this physiological response is crucial for phosphate regulation during early stages of CKD, prolonged elevation in advanced stages may lead to adverse effects [9].

This study aimed to assess the effects of OL-HDF on laboratory markers of mineral bone dynamics including calcium-phosphorus homeostasis, iPTH, FGF-23, soluble Klotho, BALP and TRAP-5b together with linear growth in children with CKD-5d.

### Patients and methods

This study was carried out at the Pediatric Dialysis Unit, Children’s Hospital, Ain Shams University over a 12-month period (between August 1, 2023, and August 1, 2024). Approval of the Research Ethics Committee, Faculty of Medicine, Ain Shams University was obtained (FWA 000017585; FMASU: MD188/2022). The study follows the guidelines and Declaration of Helsinki. Informed consent prior to recruitment was obtained from patients’ caregivers. Sample size was calculated in the Community Medicine Department at Ain Shams University using Power Analysis and Sample Size Software (PASS 11) (Version 11.0.08) setting power at 99% and alpha error at 5%, and assuming a dropout rate of 10%. It is estimated that a sample size of at least 30 patients with CKD on conventional HD would be enough to achieve study objectives. Hence, thirty-one children with CKD-5d aged 16 years or less who were stable on maintenance HD for at least 3 months were recruited. Patients with unsuitable vascular access blood flow, failed kidney graft, bone disorders other than CKD MBD, on medication affecting bone metabolism as steroids and patients expected not to complete the 12-month follow-up period were excluded. All patients underwent a baseline evaluation that included data regarding age, sex, cause of CKD, duration of HD therapy in months, residual urine volume, detailed musculoskeletal history, drug history including vitamin D, calcium, phosphate binders, calcimimetics and growth hormone, and surgical history mainly of fractures and orthopedic procedures. The frequency of intradialytic hypotension episodes was retrospectively studied in a six-month period before shifting to OL-HDF (while on HD) and compared to two successive six-month periods after switching to OL-HDF. A comprehensive clinical examination that included an assessment of the patient's anthropometric measurements (expressed as a z-score), gait, and presence of any skeletal deformities was carried out.

Baseline blood sampling of 10 ml was collected before a mid-week session from a peripheral vein away from the limb having the arteriovenous fistula while the patients were on conventional HD for calcium-phosphorus homeostasis (Ca, PO_4_, serum albumin, alkaline phosphatase), CRP, iPTH, BALP, TRAP-5b, soluble Klotho and FGF-23 (by ELISA techniques supplied by BT lab (501 Changsheng S Rd, Nanhu Dist, Jiaxing, Zhejiang, China)). Thereafter all selected patients were shifted to post-dilution OL-HDF using Fresenius Polysulfone dialyzers and hemodiafiltration blood lines and followed up for 1 year. At the end of the 12-month OL-HDF treatment period, the same laboratory workup was repeated.

Our study outcomes were: (1) Effect of OL-HDF on routine CKD MBD biomarkers; (2) Effect of OL-HDF on FGF-23, Klotho and bone turnover biomarkers; and (3) Effect of OL-HDF clinically on dry weight, height and z-scores, and change of medication doses.

Analysis of data was performed on an IBM personal computer, using Statistical Program for Social Science version 26, (SPSS Inc., Chicago, IL, USA). Data were represented as number and percentage for qualitative variables, mean ± standard deviation (*x̄* ± SD) for parametric variables, and median and interquartile range (IQR) for non-parametric variables. The Shapiro–Wilk test was applied to test for normality, paired t-test for comparison between parametric dependent variables and Wilcoxon Signed Rank test for non-parametric dependent variables. Comparison of qualitative variables was done using Chi-square (x^2^) including Pearson's chi-square test and Fisher's exact test. Correlations were studied using Spearman rho test. P value of < 0.05 was considered significant in all analyses.

## Results

We included 31 children (17 males) with an age of 12.5 (IQR = 9.7–13.25) years and an HD vintage of 60.1 (IQR = 9.1x-37.5) months. Flow chart and demographics of the study population at baseline are shown in Supplementary Fig. 1 and Supplementary Table 1. All recruited patients (100%) completed 12-month follow-up duration on post-dilution OL-HDF without any dropouts. Twenty-three (74.2%) patients had convection volume more than 12 L/m^2^ and 8 (25.8%) patients had convection volume less than 12 L/m^2^.

### Effect of OL-HDF on routine CKD MBD biomarkers

OL-HDF resulted in significant increase in serum albumin (*p* < 0.0001), serum alkaline phosphatase (*p* = 0.004) and iPTH (*p* = 0.011). On the other hand, it did not exert any significant changes in albumin corrected serum Ca^2+^ (*p* = 0.914), serum phosphate (*p* = 0.337) and Ca x P product (*p* = 0.118). These results are shown in Supplementary Table 2.

### Effect of OL-HDF on FGF-23, Klotho and bone turnover biomarkers

**Fig. 1:**
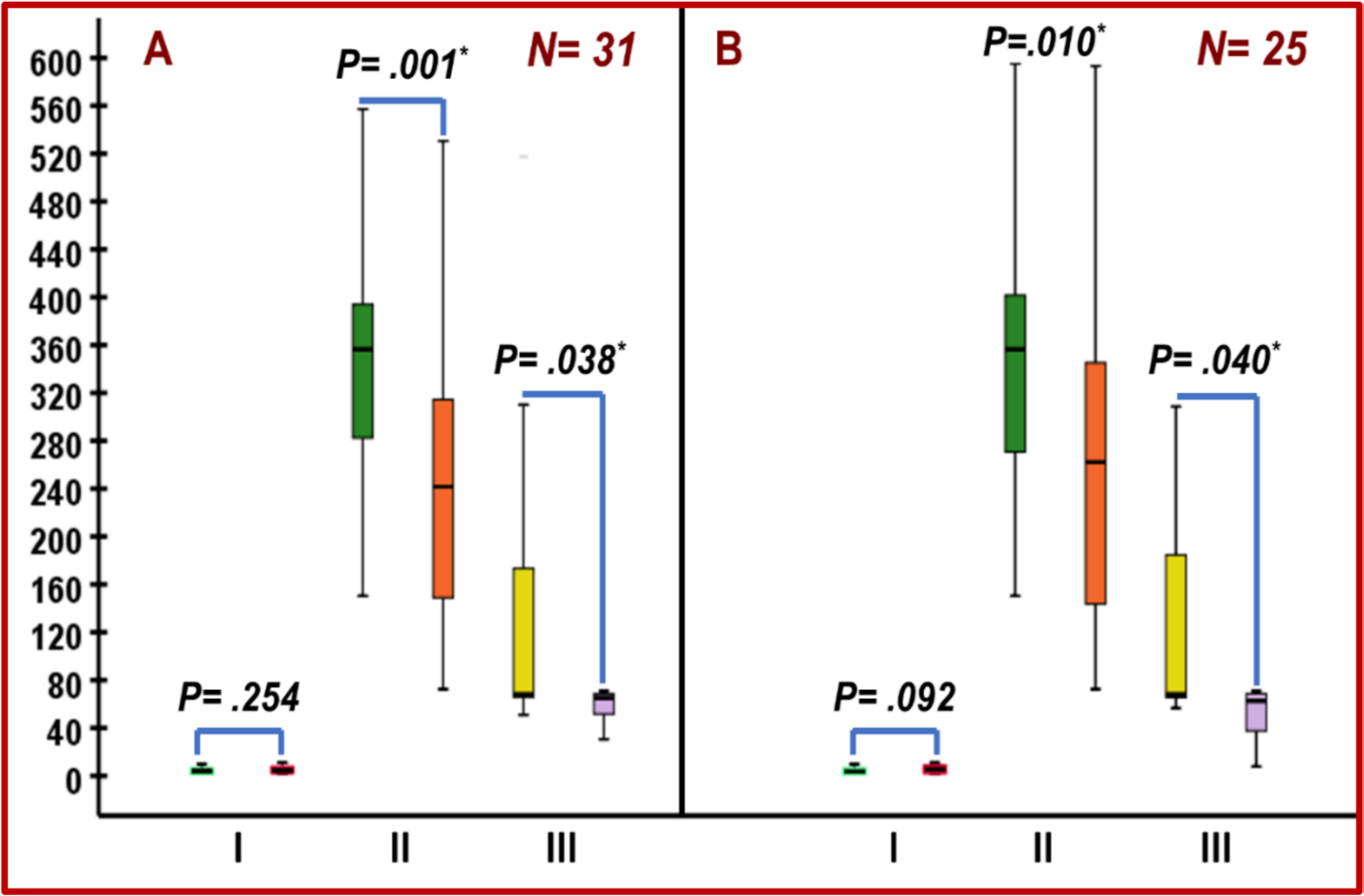
Impact of OL-HDF on Klotho and FGF-23. **A**: whole cohort (*n* = 31); **B**: children dialyzed at baseline with high flux dialyzers (*n* = 25). I: Klotho (Basal vs. at 12 months); II: FGF-23 (Basal vs. at 12 months); III: FGF-23/Klotho. (Basal vs. at 12 months); OL-HDF, online hemodiafiltration; *, P is significant

OL-HDF resulted in significant decrease in FGF-23, and FGF-23/Klotho either in the whole cohort (*n* = 31) (*p* = 0.001, *p* = 0.038, respectively) or in the subgroup dialyzed at baseline with high flux dialyzers (HFHD) (i.e. after exclusion of those on LFHD) (*n* = 25) (*p* = 0.010, *p* = 0.040, respectively). Although it increased the levels of Klotho, the increase was not significant either in the whole cohort (*n* = 31) (p = 0.254) or in the HFHD subgroup (*n* = 25) (*p* = 0.092) (Table 1 and Fig. 1).

**Table 1:** Serum level of Klotho, FGF-23, BALP and TRAP-5b in the whole cohort and high flux dialyzer hemodialysis group at baseline and after 12 months of online hemodiafiltration

	*Markers*	*Median (interquartile range)*	*P value*
*Whole cohort (n* = *31)*	Basal Klotho	4.1 ng/ml; (2.3—5.4)	***.254***
	Klotho at 12 months	4.43 ng/ml; (3.0—7.1)	
	Basal FGF-23	356.4 pg/ml (277.1—401.6)	.001^*^
	FGF-23 at 12 months	241.6 pg/ml (143.5—322.2)	
	Basal FGF-23/Klotho	68.2 (65.4—184.6)	.038^*^
	FGF-23/Klotho at 12 months	65.1 (48.4—68.8)	
	Basal BALP	100.4 IU/L; (87.7—111.8)	.007^*^
	BALP at 12 months	136.6 IU/L; (86.3–164.8)	
	Basal TRAP-5b	5.5 U/L (4.3–9.0)	.001^*^
	TRAP-5b at 12 months	3.6 U/L (3.0–5.1)	
	Basal BALP/TRAP-5b	20.0 (10.8–20.7)	< .0001^*^
	BALP/TRAP-5b at 12 months	23.9 (20.7–43.4)	
*High flux dialyzer hemodialysis patients (n* = *25)*	Basal Klotho	3.7 ng/ml; (2.3—5.4)	***.092***
	Klotho at 12 months	5.4 ng/ml; (3.0—8.3)	
	Basal FGF-23	356.4 pg/ml (261.4—451.6)	.010^*^
	FGF-23 at 12 months	262.2 pg/ml (132.7—347.4)	
	Basal FGF-23/Klotho	68.2 (65.3—192.7)	.040^*^
	FGF-23/Klotho at 12 months	62.62 (36.4—68.8)	
	Basal BALP	99.0 IU/L; (87.6—110.5)	.004^*^
	BALP at 12 months	143.2 IU/L; (90.7–168.9)	
	Basal TRAP-5b	5.5 U/L (4.4–9.1)	.004^*^
	TRAP-5b at 12 months	3.6 U/L (2.9–5.1)	
	Basal BALP/TRAP-5b	20.1 (10.6–20.8)	< .0001^*^
	BALP/TRAP-5b at 12 months	24.8 (20.9–46.5)	

^*^, P value is significant

**Fig. 2:**
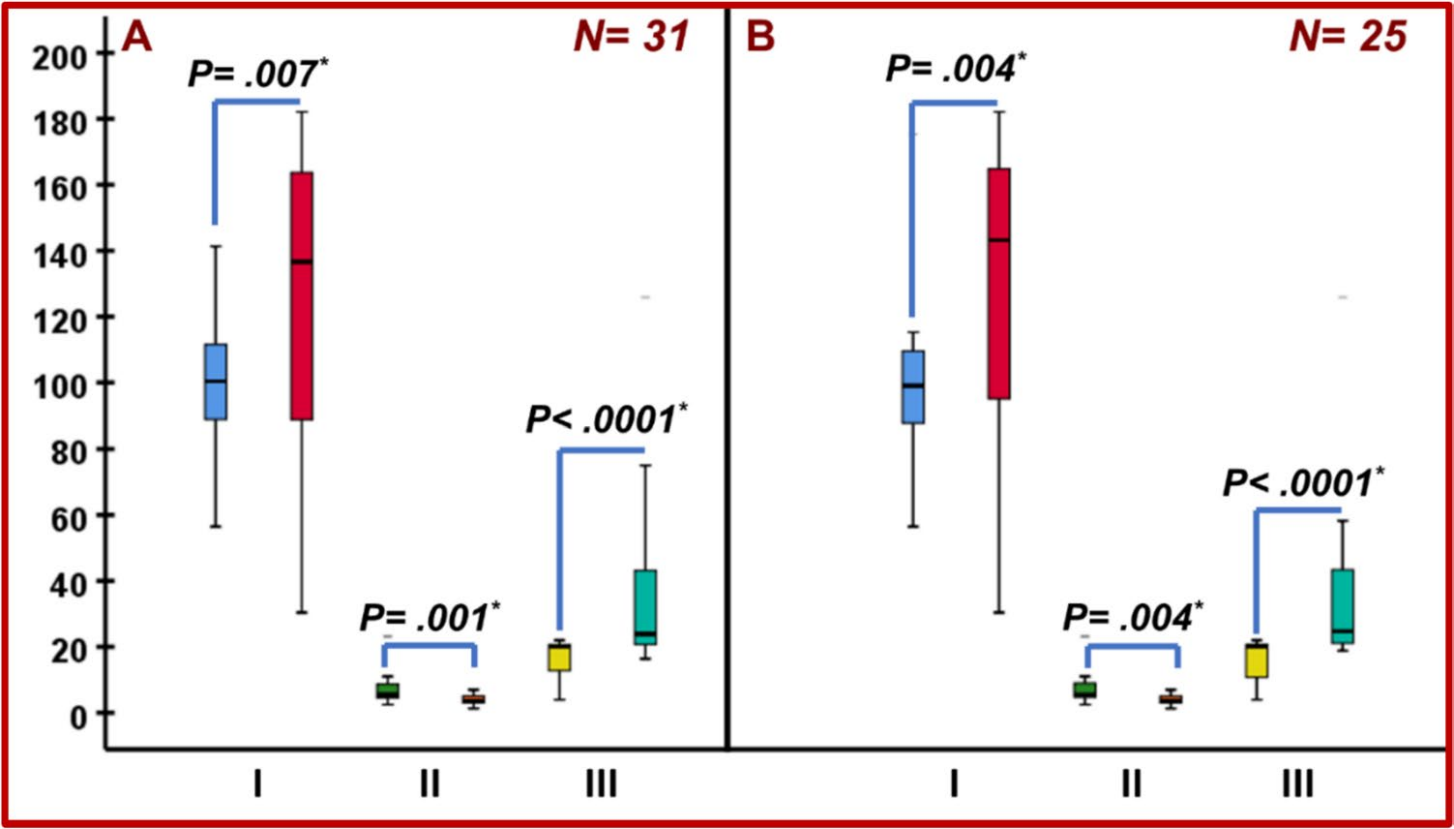
Impact of OL-HDF on BALP and TRAP-5b. **A**: whole cohort (*n* = 31); **B**: children dialyzed at baseline with high flux dialyzers (*n* = 25). I: BALP (Basal vs. at 12 months); II: TRAP-5b (Basal vs. at 12 months); III: BALP/TRAP-5b. (Basal vs. at 12 months); OL-HDF, online hemodiafiltration; *, P is significant

Also, OL-HDF resulted in significant decrease in TRAP-5b and increase in both BALP and BALP/TRAP-5b ratio in all patients (*n* = 31) (*p* = 0.007, *p* = 0.001, *p* < 0.0001, respectively) and in the HFHD subgroup (*n* = 25) (*p* = 0.004, *p* = 0.004, *p* < 0.0001, respectively) (Table 1 and Fig. 2).

We got similar results after excluding the 8 patients having convective volume less than 12 L/m^2^ (*n* = 23). OL-HDF resulted in significant decrease in FGF-23 and FGF-23/Klotho (*p* = 0.011, *p* = 0.024, respectively) but with non-significant increase in Klotho level (*p* = 0.011). It also resulted in significant decrease in TRAP-5b and increase in both BALP and BALP/TRAP-5b ratio (*p* = 0.004, *p* = 0.001, *p* = 0.001, respectively) as shown in Supplementary Table 3. Considering the subgroup of patients having convective volume more than 12L/m^2^ and dialyzed at baseline with high flux membrane (*n* = 18), OL-HDF resulted in significant decrease in FGF-23 and FGF-23/Klotho (*p* = 0.010, *p* = 0.028, respectively) but with non-significant increase in Klotho level (p = 0.068). It also resulted in significant decrease in TRAP-5b and increase in both BALP and BALP/TRAP-5b ratio (*p* = 0.002, *p* = 0.008, *p* = 0.002, respectively) as shown in Supplementary Table 4.

### Interplay between different biomarkers

**Fig. 3:**
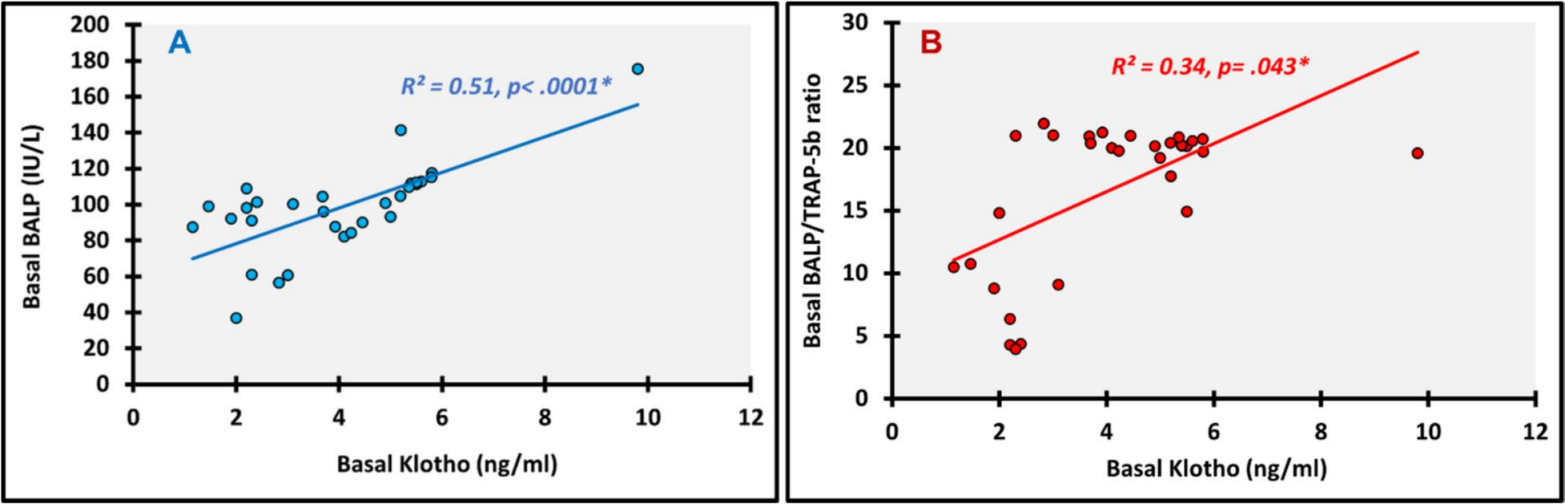
Correlations of basal Klotho. **A**: with Basal BALP; **B**: with Basal BALP/TRAP-5b ratio; *, P is significant

**Fig. 4:**
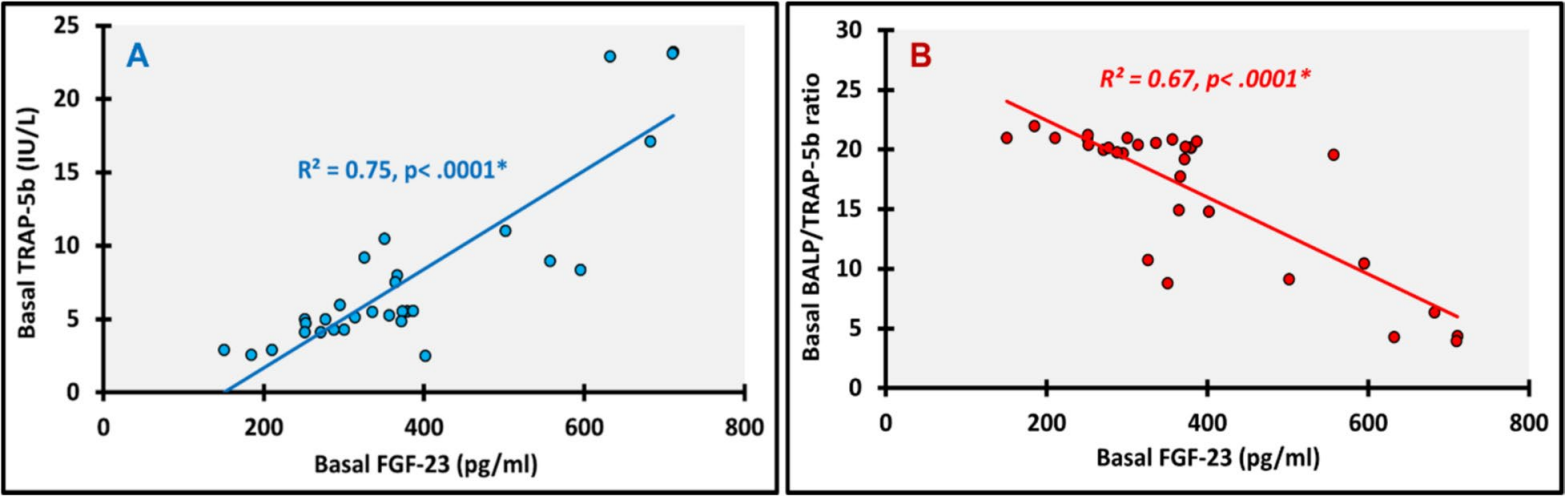
Correlations of basal FGF-23. **A**: with Basal TRAP-5b; **B**: with Basal BALP/TRAP-5b ratio; *, P is significant

**Fig. 5:**
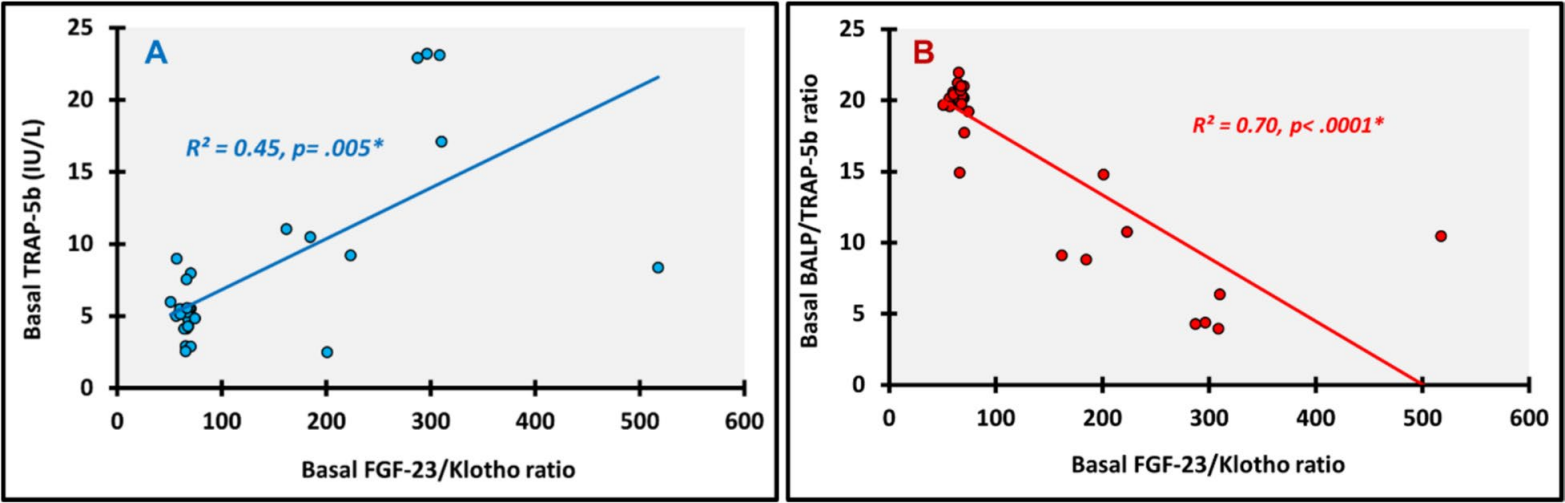
Correlations of basal FGF-23/Klotho ratio. **A**: with Basal TRAP-5b; **B**: with Basal BALP/TRAP-5b ratio; *, P is significant

**Fig. 6:**
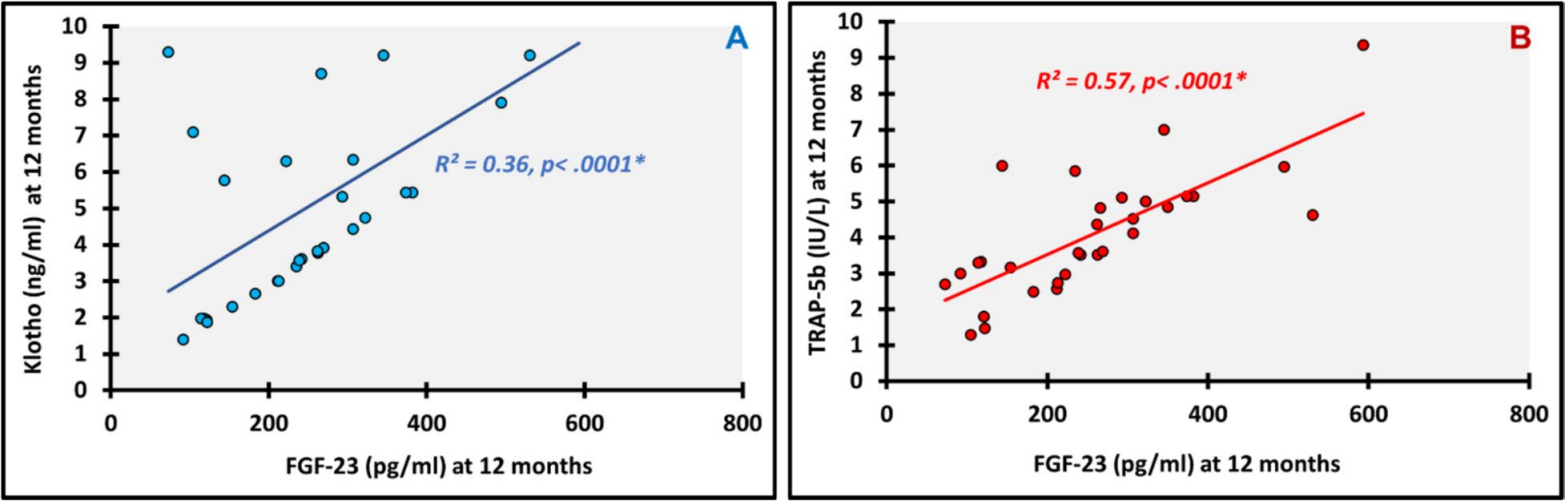
Correlations of FGF-23 at 12 months. **A**: with Klotho at 12 months; **B**: with TRAP-5b at 12 months; *, P is significant

We looked for all possible correlations within and in between the FGF-23 and Klotho profiles and bone turnover markers at baseline and at 12 months. The following are the most relevant significant correlations. At baseline Klotho showed significant positive correlation with BALP (ρ(rho) = 0.719; *p* < 0.0001) (Fig. 3A) and BALP/TRAP-5b (ρ(rho) = 0.366; *p* = 0.043) (Fig. 3B), and TRAP-5b had significant positive correlation with FGF-23 (ρ(rho) = 0.755; *p* < 0.0001) (Fig. 4A) and FGF-23/Klotho (ρ(rho) = 0.490; *p* = 0.005) (Fig. 5A). Meanwhile, significant negative correlations were detected between BALP/TRAP-5b and each of FGF-23 (ρ(rho) =—0.747; *p* < 0.0001) (Fig. 4B) and FGF-23/Klotho (ρ(rho) =—0.660; *p* < 0.0001) (Fig. 5B). At 12 months, we found significant positive correlations between FGF-23 and each of Klotho (ρ(rho) = 0.601; *p* < 0.0001) (Fig. 6A) and TRAP-5b (ρ(rho) = 0.759; *p* < 0.0001) (Fig. 6B).

Multiple linear regression model analyses were done and the following are the most significant models found. A multiple linear regression was run to predict basal BALP based on basal Klotho and basal FGF-23 and resulted in a significant model (F = 32.006, p < 0.0001, R^2^ = 0.696). Predicted BALP = 27.27 + 10.824 (Klotho) + 0.072 (FGF-23). Both Klotho and FGF-23 were significant predictors (t = 7.412, *p* < 0.0001; t = 4.136, *p* < 0.0001) but Klotho was the predominating independent variable. A multiple linear regression was run to predict basal TRAP-5b based on basal Klotho and basal FGF-23 and resulted in a significant model (F = 52.695, *p* < 0.0001, R^2^ = 0.790). Predicted TRAP-5b = −2.005–0.638 (Klotho) + 0.032 (FGF-23). Both Klotho and FGF-23 were significant predictors (t = −2.247, *p* = 0.033; t = 9.542, *p* < 0.0001) but FGF-23 was the predominant independent variable. A multiple linear regression was run to predict basal BALP/TRAP-5b ratio based on basal Klotho and basal FGF-23 and resulted in a significant model (F = 106.314, *p* < 0.0001, R^2^ = 0.884). Predicted BALP/TRAP-5b = 21.559 + 1.532 (Klotho)—0.029 (FGF-23). Both Klotho and FGF-23 were significant predictors (t = 7.155, *p* < 0.0001; t = −11.428, *p* < 0.0001) but FGF-23 was the predominant independent variable. The relations between basal BALP, basal TRAP-5b and basal BALP/TRAP-5b ratio, and their predicted values are shown in Supplementary Figs. 2, 3, and 4.

There were no significant correlations between Klotho, FGF-23, FGF-23/Klotho, BALP, TRAP-5b and BALP/TRAP-5b with age, height, residual urine volume, blood pump flow rate, convective volume, spKt/V, doses of required medications (alfacalcidol), oral calcium, phosphate binder (sevelamer), calcimimetics (cinacalcet), serum albumin, corrected serum Ca2 +, serum phosphate, and serum iPTH at any point. Generalized linear modeling did not reveal any significant effect of gender, age group (< 10 years and ≥ 10 years), presence of bony aches, deformities and gait abnormality, patient’s compliance, HD duration (< 1 year and ≥ 1 year), and the need for alfacalcidol, oral calcium, phosphate binder (sevelamer) and calcimimetics (cinacalcet) on Klotho, FGF-23, FGF-23/Klotho, BALP, TRAP-5b and BALP/TRAP-5b as dependent variables at any point.

#### Clinical outcomes

After a 12-month treatment period of OL-HDF, weight z-score was significantly lower than baseline values (*p* < 0.0001) but height z-score was not significantly changed, either before or after exclusion of patients with deformities (*p* = 0.130, *p* = 0.153, respectively) (Supplementary Table 5; Supplementary Figs. 5 and 6).

We took the number of episodes of intradialytic hypotension (IDH) as an indicator for the tolerability of OL-HDF. The occurrence of IDH during the last 6 months of HD before shifting to OL-HDF was 0.5 episode/patient/session that dropped to 0.1 episode/patient/session and to 0.03 episode/patient/session during the first and second 6 months of OL-HDF treatment, respectively. The number of IDH episodes were [Mdn (IQR)] 40.0 (20.0–60.0), 0.0 (0.0–20.0), and 0.0 (0.0–6.0) during the last 6 months of HD before shifting to OL-HDF and during the first and second 6 months of OL-HDF treatment respectively. The number of IDH episodes was significantly lower during the first and second 6 months of OL-HDF treatment than during the last 6 months of HD (*p* < 0.0001; *p* < 0.0001, respectively). It was also significantly lower during the second 6 months of OL-HDF compared to the first 6 months (*p* = 0.002).

Although puberty stage was not evaluated, we made up for it by considering the age of 13 years as a cut-off to assess the effect of HD and OL-HDF on the changes in height SDS. However, we did not find any significant difference in height SDS in children above 13 years at baseline (*n* = 10) and at 12 months (*n* = 18) (*p* = 0.460).

Regarding the need for supplementary medication, OL-HDF therapy did not show any significant changes in the percent of cases who needed oral calcium, alfacalcidol, phosphate binder (sevelamer), and calcimimetics (cinacalcet) or in the doses of these medications. These results are shown in Supplementary Table 6.

## Discussion

Significant alterations in mineral and bone metabolism are known in CKD and are prominent factors in morbidity and poor quality of life. OL-HDF is a superior dialysis technique that has been established since 1978 and has proved its effectiveness in removal of medium-weight molecules including beta-2 microglobulin (β2M), which is the prototype of medium-toxin clearance [10].

Our study confirmed the effect of OL-HDF in lowering FGF-23 levels significantly in children who were on regular conventional HD after being shifted to post-dilution OL-HDF for 12 months compared to their baseline level. Even among the subgroup of HD patients who used high-flux dialyzers, this reduction was still statistically significant. Supporting our findings, Fischer et al. reported that FGF-23 levels decreased by 25% in children on post-dilution OL-HDF with polysulphone dialyzers but increased by 100% in those on conventional HD with high flux polysulphone dialyzers, resulting in a significant difference between the two groups at 12 months. Similar results were reported by other studies [11, 12]. Uhlin et al. reported different findings. They found that FGF-23 levels were unaltered at any follow-up time points (6, 12 and 24 months) following the switch from HD to OL-HDF. This may be due to many dropouts during their study, as it started with 35 patients and ended up with 11 patients only [13].

FGF-23 expression is markedly increased in acute inflammation; however, osteocytes can counteract this increase in FGF-23 transcription by increasing FGF-23 cleavage, thus keeping serum levels of biologically active FGF-23 normal. In contrast, chronic inflammation associating uremia raises biologically active intact FGF-23 levels because prolonged periods of FGF-23 overproduction exceed the capacity of the FGF-23 breakdown in osteocytes [14]. Studying cytokines in a cohort of adult CKD patients has shown that many of them may increase the production of FGF-23 [14]. Furthermore, inflammation may also increase the production of FGF-23 indirectly through hepcidin activation and hypoferremia [15]. Thus, lower serum levels of FGF-23 by HDF can be explained by the fact that intact FGF23 has a molecular weight of 31 kDa and is cleared by HDF [12]. Also, it might be due to reduced FGF23 production as HDF is more efficient in clearance of large middle-sized molecules, including proinflammatory cytokines and other uremic toxins [11].

Changes in FGF-23 levels in our study were independent of serum phosphate, which did not change after 12 months of OL-HDF therapy. Additionally, we did not find any significant correlation between the levels of these 2 variables at any point of time. Fischer et al. also reported that the changes in FGF-23 were independent of serum phosphate [11]. On the contrary, Lima et al*.* found significant positive correlation between FGF-23 and serum phosphate [16]. The difference is probably because in the latter study patients were on HD and not HDF.

High FGF-23, low Klotho levels, and high FGF-23/Klotho ratio in CKD and HD adults are noted by many studies [17]. But, to our knowledge, the effect of HDF on Klotho and FGF-23/Klotho has been studied by only one other study [11]. We did not find any significant difference in Klotho between basal levels and after 12 months of OL-HDF, either within all patients group or the subgroup of HD patients who used high flux dialyzers. The same result was found by Fischer et al. [11]*.* As a result of decreased FGF-23 and unaltered Klotho, the level of FGF-23/Klotho ratio significantly decreased after OL-HDF as compared to the baseline level within all patients group and those who received HD using high flux dialyzers. Similarly, Fischer et al. found that the FGF-23/Klotho ratio was significantly reduced in children on post-dilution OL-HDF with polysulphone membranes compared to children on conventional HD using high flux polysulphone dialyzers after 12 months of therapy. They concluded that changes in this ratio were dependent mainly on dialysis modality and inflammatory markers levels [11].

While bone turnover is best diagnosed by bone biopsy, serum BALP levels, reflective of bone formation, and TRAP-5b, of bone resorption, are useful surrogate measures [18]. Unlike other bone biomarkers, BALP and TRAP-5b are both unaffected by CKD stage [18]. Our study revealed that 12 months of post-dilution OL-HDF treatment resulted in significant increase in BALP levels within all patient groups and the subgroup of patients receiving conventional HD using high flux dialyzers. Fischer et al*.* found that BALP increased in children on post-dilution OL-HDF with polysulphone membranes but decreased in children on conventional HD with high flux polysulphone dialyzers, with significantly raised levels in OL-HDF as compared to HD patients at 12 months; similar findings were mentioned in another study [11, 19]*.* On the other hand, another study showed no statistically significant difference in BALP after 3 months on post-dilution OL-HDF[20]*.* Small sample size and short duration of follow-up might explain the different results of this study.

Our study also revealed that 12 months of post-dilution OL-HDF treatment resulted in significant decrease in TRAP-5b levels within all patient groups and the subgroup of patients receiving conventional HD using high flux dialyzers. Fischer et al. showed increased TRAP-5b in children on conventional HD with high flux polysulphone dialyzers over the 12-month follow-up period but remained unchanged in those on post-dilution OL-HDF with polysulphone membranes. Despite this, TRAP-5b was significantly lower in OL-HDF compared with HD at 12 months [11]. We found that 12-month duration of post-dilution OL-HDF treatment resulted in significant increase in the BALP/TRAP-5b ratio compared to basal values within all patient groups and the subgroup of patients who were on conventional HD using high flux dialyzers. Fischer et al. found significant increase in the BALP/TRAP-5b ratio in children on post-dilution OL-HDF with polysulphone membranes but remained unchanged in those on conventional HD with high flux polysulphone dialyzers over a year of follow-up, showing a significant higher level in the OL-HDF in comparison to HD [11]. Our results and those of Fischer et al.showed that children on OL-HDF had a BALP/TRAP-5b ratio that was statistically comparable to normal children, implying an osteoanabolic process [18].

In healthy children BALP and TRAP-5b are strongly positively correlated with each other [18]. In our study we did not find any correlation between these 2 markers. Meanwhile, Fischer et al. found that BALP and TRAP-5b were weakly correlated in children on HD and OL-HDF, suggesting that there might be uncoupling of bone formation and resorption. The changes induced by OL-HDF in BALP and TRAP-5b, and hence in the BALP/TRAP-5b ratio might be related to reduction in the inflammatory milieu. The BALP/TRAP-5b ratio was inversely correlated with the inflammatory markers IL-6, TNF-α, and high sensitivity CRP [11]. Another suggested explanation is due to FGF-23 changes which have an inhibitory effect on bone mineralization, but whether they act directly or indirectly is not yet fully understood. Inhibition of 25-hydroxyvitamin D-1α-hydroxylase, inhibition of BALP, upregulation of TRAP-5b and induction of inflammatory markers were proposed [21]. Our findings of positive correlations between FGF-23 and TRAP-5b basally and after 12 months of follow-up, negative correlation between the basal BALP/TRAP-5b ratio and each of basal FGF-23 and basal FGF-23/Klotho and the predominating effect of basal FGF-23 over basal Klotho as independent predictors for basal BALP/TRAP-5b ratio might be suggested as supporting points for this explanation.

Like our findings, Fischer et al*.* did not find any associations or relation between dialysis modality, residual kidney function (urine output in ml/kg/h), medications, FGF-23, Klotho, or bone turnover biomarkers [11].

Our study showed a significant decrease in mean value of dry weight z-score despite the increase in the mean value of dry weight after 12 months of post-dilution OL-HDF treatment*.* One study held in our center found that there was no significant difference in weight SDS between children on OL-HDF and those on HD at 3, 9 and 18 months of follow-up. However, the study showed that children on OL-HDF had significantly higher percent changes of weight SDS at intervals between 3 and 9 months and between 3 and 18 months compared to the HD group [22]. We think that the increase in the absolute values of the dry weights of our patients after 12 months of OL-HDF was not reflected on the z-scores of their dry weight because many patients tolerated decreasing their dry weight to the optimum after being shifted to OL-HDF. OL-HDF can potentially improve nutritional status in patients by enhancing the removal of uremic toxins and reducing inflammation, which can lead to better appetite and less protein-energy wasting. This is reflected by the significant increase in serum albumin in our children after being shifted to OL-HDF. However, it must be considered that our sample size is relatively small and probably a longer period is needed to have proper catch-up of their dry weight to be positively reflected on dry weight z-score.

The significant decrease in the episodes of IDH during OL-HDF treatment compared to conventional HD reflects that our children tolerated OL-HDF better than conventional HD. In our study the convection volume achieved was [Mdn (IQR)] 13.2 (11.9–14.5) L/m^2^ which is comparable to that reported by Shroff et al. [13.3; (11.5—14.2) L/m^2^] [23]. After 12 months of post-dilution OL-HDF, significant increase in serum albumin, serum alkaline phosphatase and iPTH was observed but it did not exert any significant changes in corrected serum Ca^2+^ and serum phosphate. Fischer et al. did not find any significant difference in the regularly measured biomarkers of CKD-MBD, including corrected serum Ca2 +, serum phosphorus and iPTH between HD and OL-HDF cohorts at baseline and at 12 months [11]. In contrast, Ibrahim et al. and Francisco et al. reported that serum phosphate level was lower in their OL- HDF cohort than HD cohort [22, 24]. The increase in the level of PTH after 12 months of OL-HDF in our study can be explained by reduced levels of FGF-23 that have an inhibitory effect on the parathyroid gland. Uhlin et al. reported that the switch from HD to OL-HDF resulted in significantly decreased serum 25(OH)D3 and hence higher PTH levels [13].

We did not find any significant changes in height z- score after 12 months of post-dilution OL-HDF treatment before and after exclusion of patients having lower limb deformities. However, one of our main study limitations is that we did not adjust for puberty assessment as a confounding variable. Improved growth in children on OL-HDF was first reported by Fischbach et al. who observed a significant improvement in growth velocity after one year of pre-dilutional OL-HDF. However, this study performed OL-HDF six times a week, and most children were treated with growth hormone, making it hard to conclude the advantages of OL-HDF alone [25]. Shroff et al. state that the annual change in height SD score remained unchanged in children on HD but observed little yet statistically significant increment in those on post-dilution OL-HDF, so that children on OL-HDF were taller than those on HD after a year of follow-up; these findings were independent of growth hormone treatment [23]. Also, Ibrahim et al. stated that children on OL-HDF had significantly increased height SDS at 3, 9, and 18 months and percent changes of height SDS at intervals between 3 and 9 months and between 3 and 18 months compared to those on HD [22]. Fischer et al. reported non-significant differences in height SDS in children on HD and OL-HDF basally. Although after one year of follow-up children on HD were shorter, there was still no significant difference in the height SDS between HD and OL-HDF patients [11]. Like our study, Shroff et al. did not assess the pubertal status of their patients, but instead, they compared the median annualized change in height SD score in children above 13 years of age between HD and HDF groups and found it was statistically significant (*p* = 0.005) [23]. Following their methods [23], we considered the age of 13 years as a cut-off to assess the effect of HD and OL-HDF on the changes in height SDS. However, we did not find any significant difference in height SDS in children above 13 years at baseline (*n* = 10) and at 12 months (*n* = 18) (*p* = 0.460).

We think that the small sample size, design of the study, the duration of follow-up, the difference in the way of expressing the result (e.g. z-score, z-score percent of change), not considering cases having deformities, and lack of assessment of pubertal changes contribute to the differences between all the previous studies and our study.

Some limitations of the current study must be acknowledged including the small sample size and lack of adjustment for puberty assessment as a confounding variable.

## Conclusion

OL-HDF resulted in a significant decrease in FGF-23, TRAP-5b and FGF-23/Klotho ratio with a significant increase in BALP and the BALP/TRAP-5b ratio. This might signify a promising positive impact of OL-HDF on the osteoanabolic process in children with CKD-5d and the need to prioritize this HD modality in them.

## Supplementary Information

Below is the link to the electronic supplementary material.

Graphical abstract (PPTX 292 KB)

Supplementary file1 (DOCX 820 KB)

Supplementary file2 (DOCX 33 KB)

## Data Availability

Data will be available upon request to the corresponding author.
