# Supplementary material for: Impact of online hemodiafiltration on bone turnover in children with CKD-5d: A prospective cohort study
Source: Pediatr Nephrol. 2025 May 19;40(10):3253–62. doi: 10.1007/s00467-025-06805-2 (PMC12401763; doi:10.1007/s00467-025-06805-2)
Supplement: Supplementary file 1 — Graphical abstract (PPTX 292 KB) [file 467_2025_6805_MOESM1_ESM.pptx]

## Slide 1
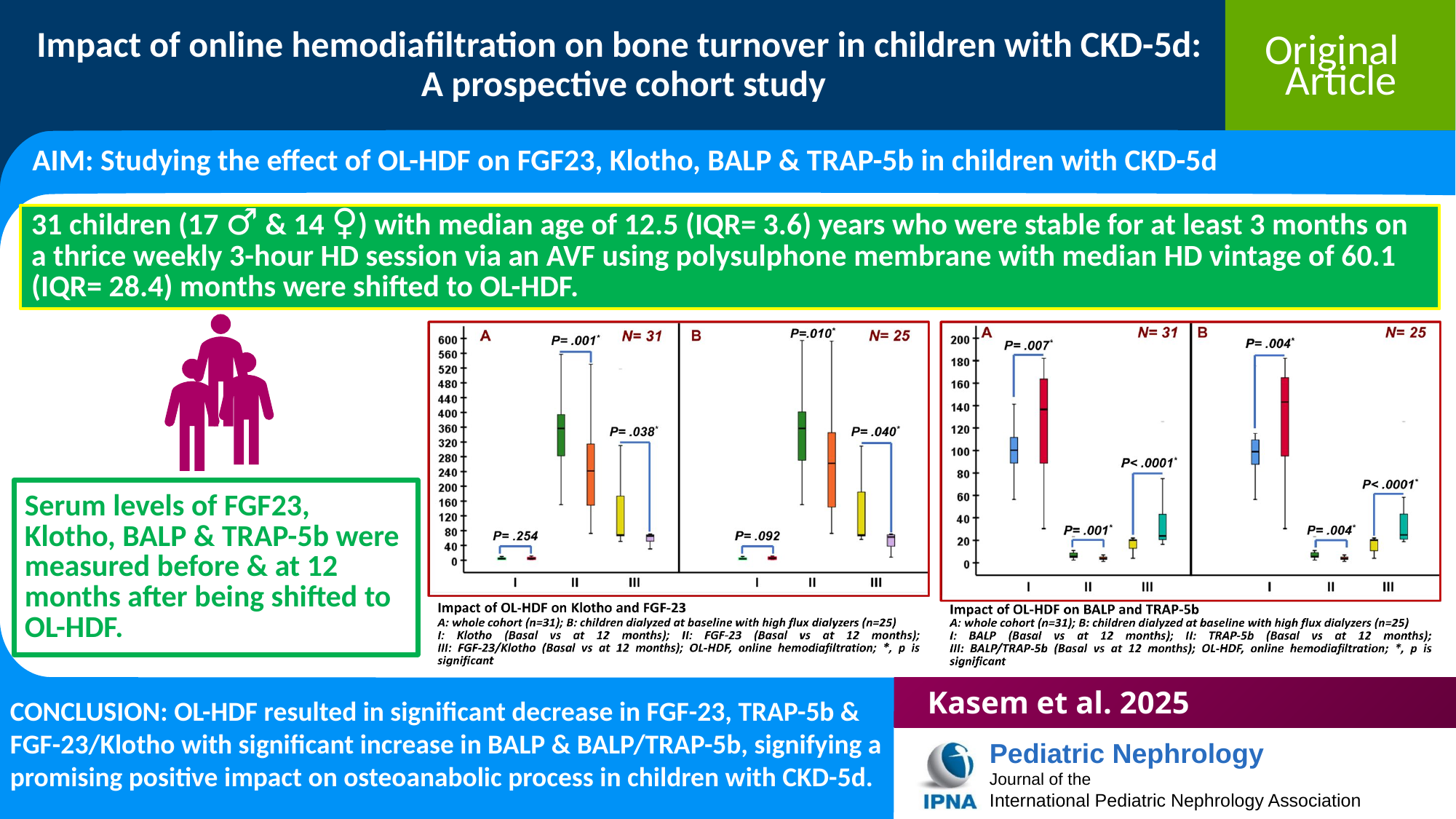

Impact of online hemodiafiltration on bone turnover in children with CKD-5d:
A prospective cohort study
AIM: Studying the effect of OL-HDF on FGF23, Klotho, BALP & TRAP-5b in children with CKD-5d
31 children (17 ♂ & 14 ♀) with median age of 12.5 (IQR= 3.6) years who were stable for at least 3 months on a thrice weekly 3-hour HD session via an AVF using polysulphone membrane with median HD vintage of 60.1 (IQR= 28.4) months were shifted to OL-HDF.
Serum levels of FGF23, Klotho, BALP & TRAP-5b were measured before & at 12 months after being shifted to OL-HDF.
Kasem et al. 2025
CONCLUSION: OL-HDF resulted in significant decrease in FGF-23, TRAP-5b & FGF-23/Klotho with significant increase in BALP & BALP/TRAP-5b, signifying a promising positive impact on osteoanabolic process in children with CKD-5d.
