# Supplementary material for: Impact of online hemodiafiltration on bone turnover in children with CKD-5d: A prospective cohort study
Source: Pediatr Nephrol. 2025 May 19;40(10):3253–62. doi: 10.1007/s00467-025-06805-2 (PMC12401763; doi:10.1007/s00467-025-06805-2)
Supplement: Supplementary file 2 — Supplementary file1 (DOCX 820 KB) [file 467_2025_6805_MOESM2_ESM.docx]

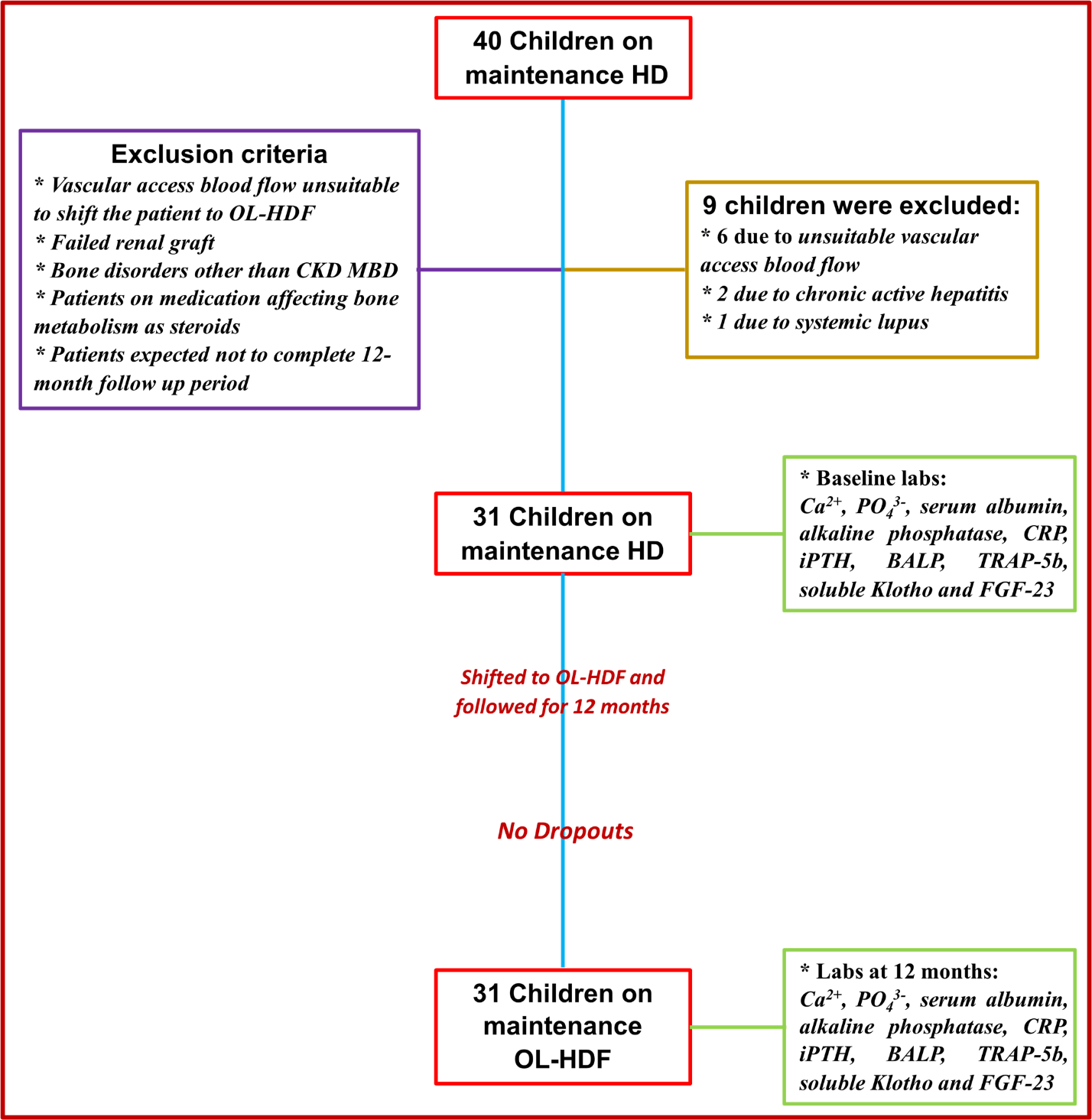
**Supplementary Figure 1. Flow chart of the study**

HD, hemodialysis; OL-HDF, online hemodiafiltration; Ca^2+^, serum calcium; PO_4_^3-^, serum phosphorus; CRP, C-reactive protein; iPTH, intact parathyroid hormone; BALP, bone specific alkaline phosphatase; TRAP-5b, tartarate resistant acid phosphatase; FGF-23, fibroblast growth factor-23

**
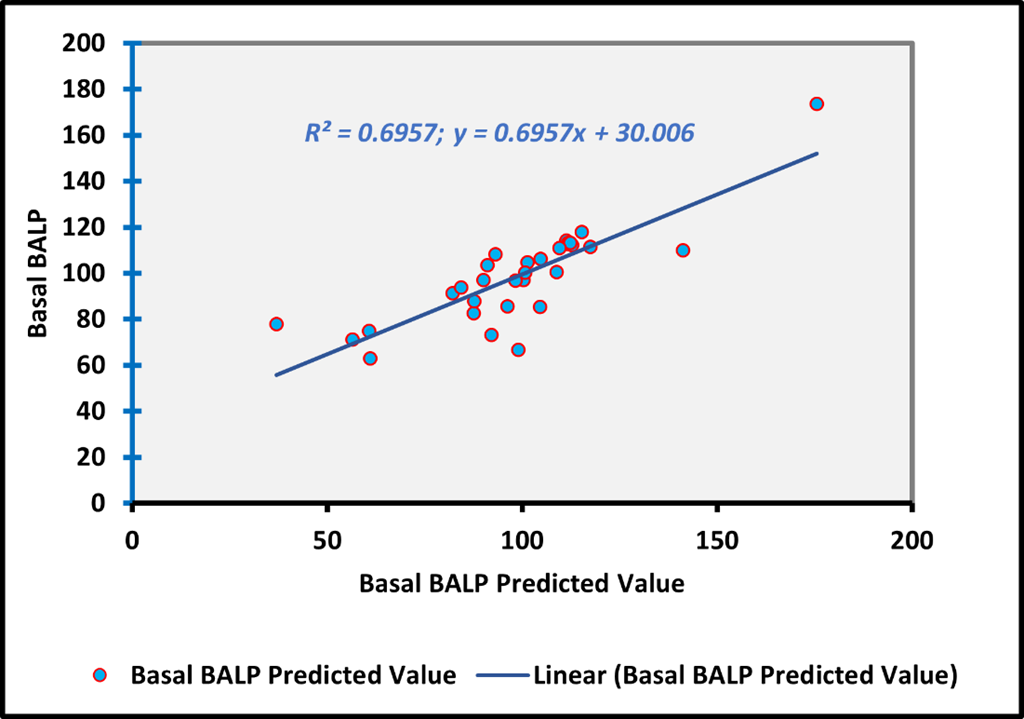
**

**Supplementary Figure 2. Relation between basal BALP and its predicted value**


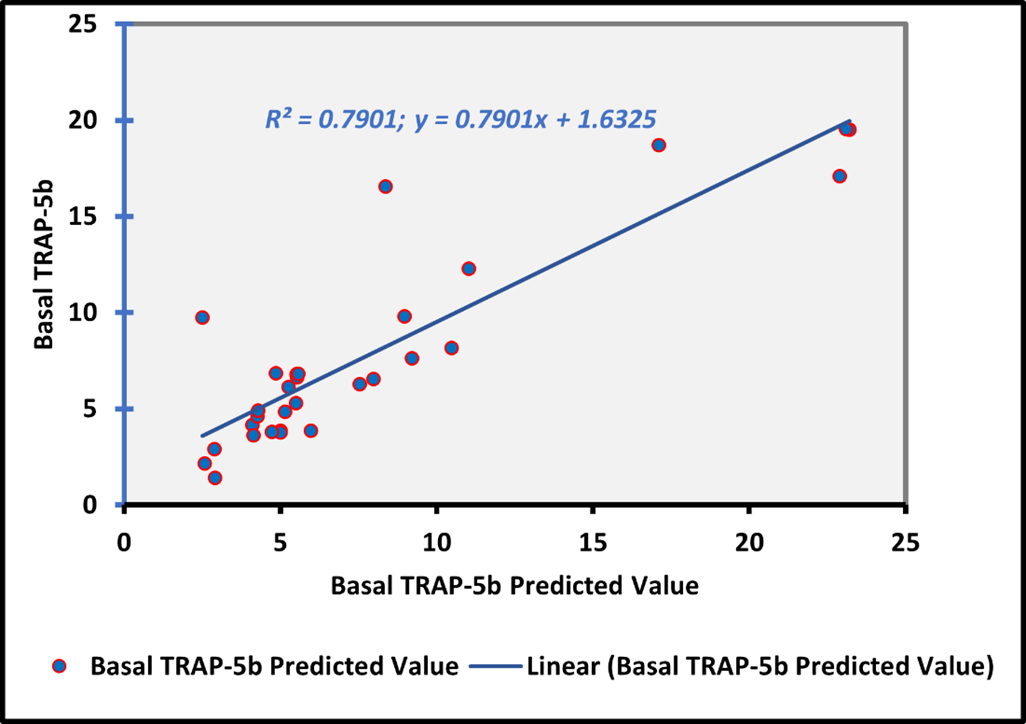


**Supplementary Figure 3. Relation between basal TRAP-5b and its predicted value**


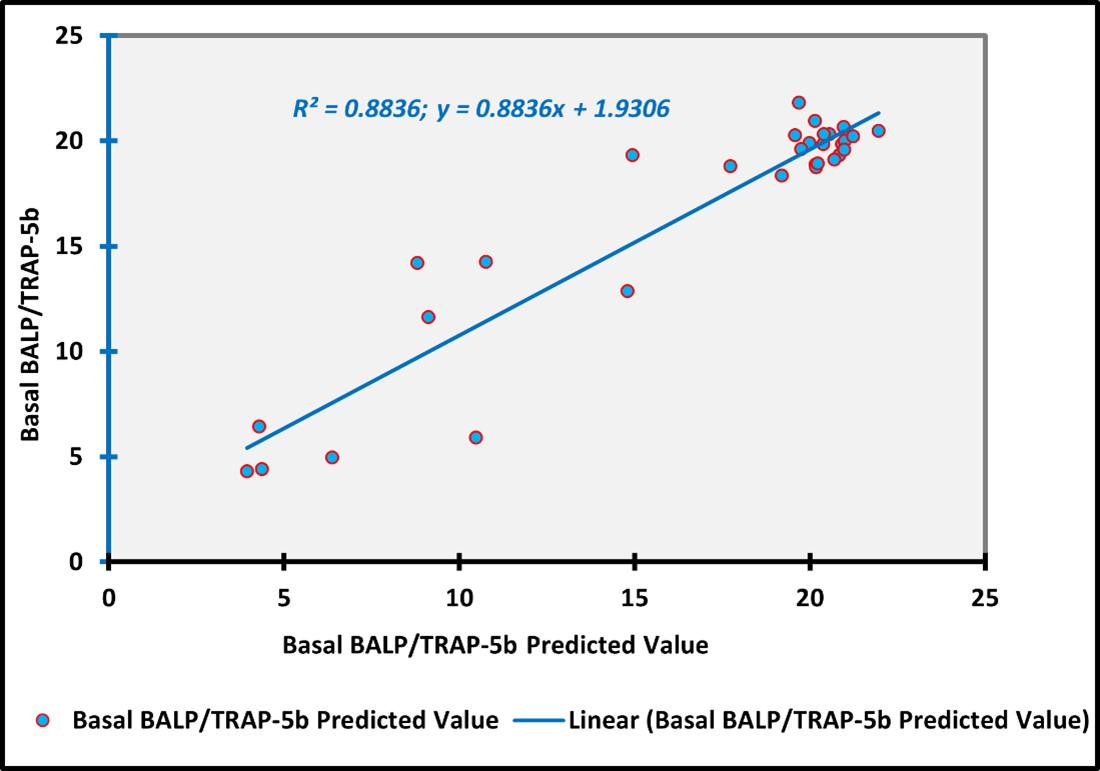


**Supplementary Figure 4. Relation between basal BALP/TRAP-5b ratio and its predicted value**


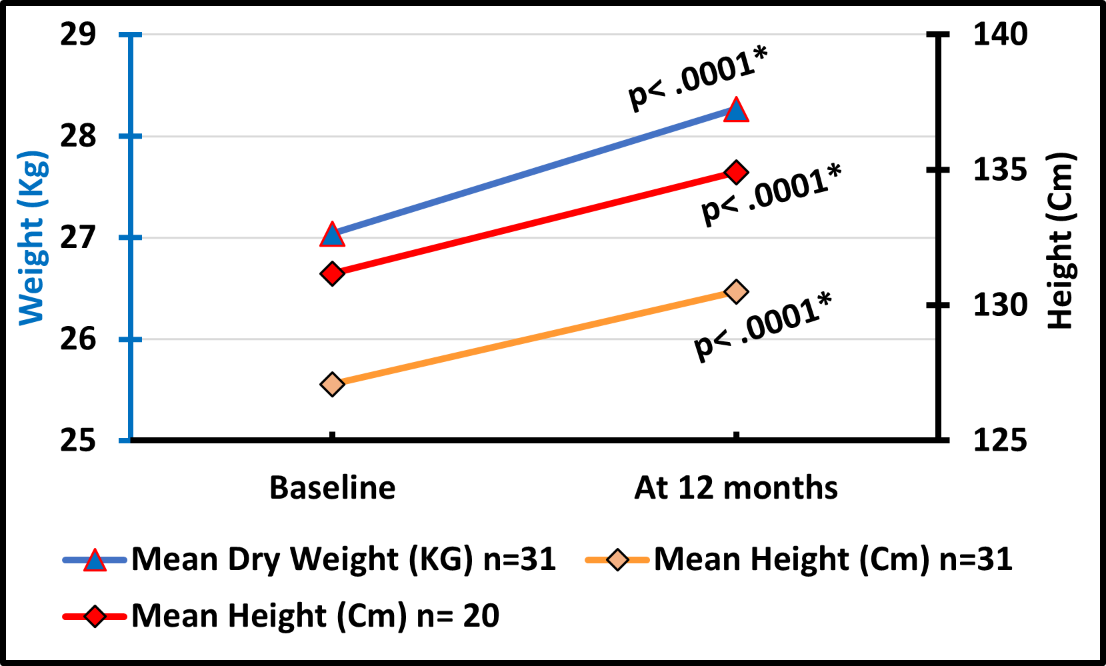


**Supplementary Figure 5. Impact of OL-HDF on mean weight and height**

n=31, the whole cohort; n=20, patients without deformities; ^*^, P value is significant

**Fig. Sf6**

**Impact of OL-HDF on weight and height Z Scores**

***n=31, the whole cohort; n=20, patients without deformities; ^*^, P value is significant***


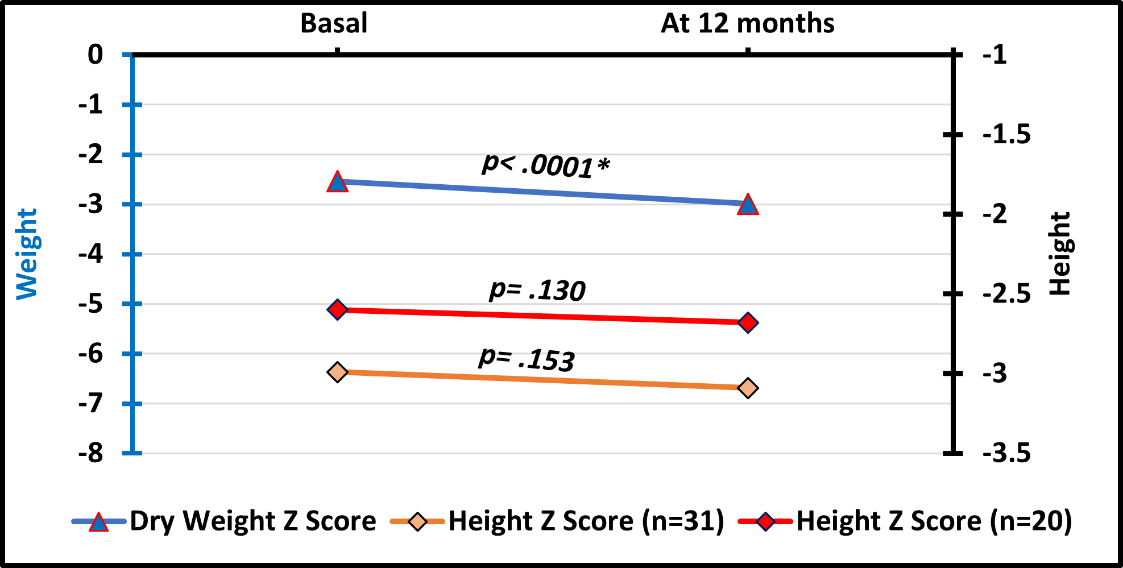


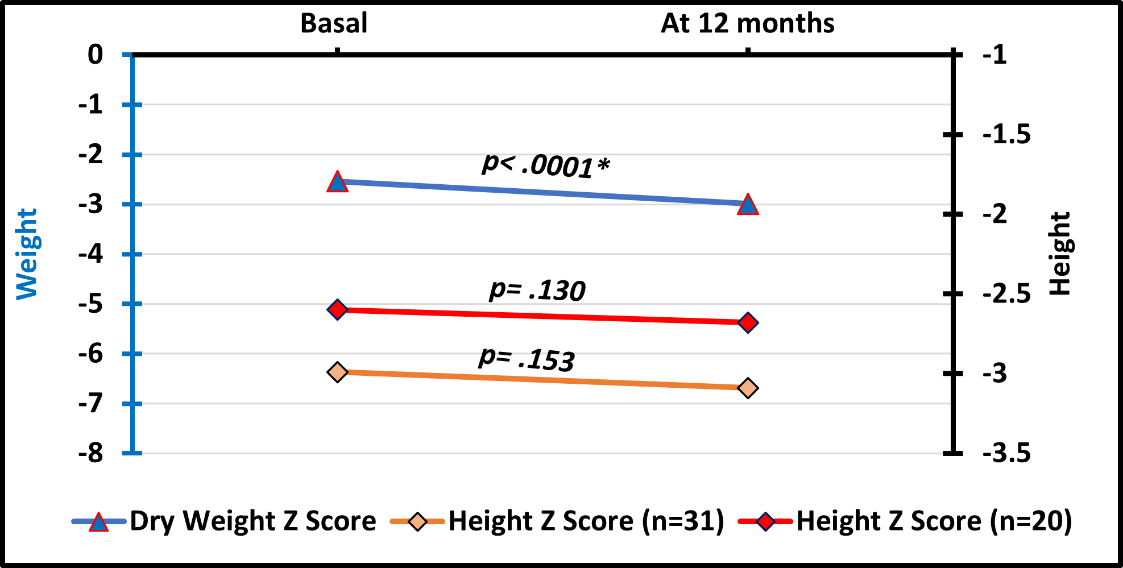


**Supplementary Figure 6. Impact of OL-HDF on weight and height Z Scores**

n=31, the whole cohort; n=20, patients without deformities; ^*^, P value is significant
