# Supplementary material for: Impact of online hemodiafiltration on bone turnover in children with CKD-5d: A prospective cohort study
Source: Pediatr Nephrol. 2025 May 19;40(10):3253–62. doi: 10.1007/s00467-025-06805-2 (PMC12401763; doi:10.1007/s00467-025-06805-2)
Supplement: Supplementary file 3 — Supplementary file2 (DOCX 33 KB) [file 467_2025_6805_MOESM3_ESM.docx]

| Parameters | Basal data |
| --- | --- |
| Number | 31 patients |
| Age at recruitment: Mdn (IQR) | 12.5 (9.7 - 13.3) years |
| Gender: n (%) | Males: 17 (55%) |
| Underlying kidney disease n (%) | 16 (52%)  6 (19%)  5 (16%)  2 (7%)  1 (3%)  1 (3%) |
| * CAKUT  * Chronic glomerulopathy  * Ciliopathies  * TMA  * Metabolic  * Unknown |  |
| HD vintage: Mdn (IQR) | 16.1 (9.1 - 37.5) months |
| Residual urine volume: n (%) | ˂0.5ml/kg/hr: 22 (71%)  ≥ 0.5ml/kg/hr: 9 (29%) |
| Bony aches (yes/no) n (%) | 20 (65%)/ 11 (35%) |
| Bone deformities (yes/ no) n (%) | 11 (35%)/ 20 (65%) |
| Medications: n (%) |  |
| Alfacalcidol | 23 (74%) |
| Calcium supplements | 20 (65%) |
| Phosphate binder (Sevelamer) | 11 (36%) |
| Calcimimitics (Cinacalcet) | 5 (16%) |
| Growth hormone | None |
| Frequency of dialysis sessions | 3 sessions/week |
| Duration of dialysis session | 3 hours/session |
| Dialyzer |  |
| * Material: n (%) | Fresenius Polysulfone: 31(100%) |
| * Performance: n (%) | LF: 6 (19%); HF: 25 (81%) |
| Blood lines | Pediatric lines |
| Dialysis machine | Fresenius 4008S, Bellco formula, Bellco formula therapy, Gambro AK 96 |
| Dialysis water quality n (%) | Ultrapure water & DIASAFE ® filter; 31(100%) |
| Vascular Access n (%) | Arterio-venous fistula (AVF) 31 (100%) |
| Blood flow: Mdn (IQR) | 230 (226 – 250) ml/minute/m^2^ |
| Source of dialysis fluid | Central Dialysis Fluid Delivery System (CDDS) |
| Dialysate flow n (%) | 500ml/minute 31 (100%) |
| Dialysis bath composition *Standard for all patients: n= 31; (100%)* | Sodium= 138 mmol/L, Potassium= 2 mmol/L, Calcium= 1.75 mmol/L, Magnesium= 0.5 mmol/L, Chloride= 106.5 mmol/L, Bicarbonate= 32 mmol/L |
| Anticoagulant n (%) | Unfractionated heparin: 31(100%) |
| spKt/V: Mdn (IQR) | 1.3 (1.2 – 1.3) |

**Supplementary Table 1. Demographics of the study population at baseline**

***CAKUT, congenital anomalies of kidney & urinary tract; TMA, thrombotic microangiopathy; HD, hemodialysis; LF, low flux; HF, high flux; Mdn, median; IQR, interquartile range***

| Parameters | Baseline | At 12 months of OL-HDF | P value |
| --- | --- | --- | --- |
| Serum albumin: x̅ ± SD | 3.9 ± 0.35 g/dL | 5.3 ± 0.27 g/dL | ***< .0001^pt*^*** |
| Albumin cCa^2+^: x̅ ± SD | 8.9 ± 1.1 mg/dL | 9.0 ± 1.3 mg/dL | ***.914 ^pt^*** |
| Serum phosphate: x̅ ± SD | 6.0 ± 1.9 mg/dL | 5.4 ± 1.7 mg/dL | ***.337 ^pt^*** |
| Ca x P by product: x̅ ± SD | 55.4 ± 18.2 mg^2^/dL^2^ | 50.0 ± 16.4 mg^2^/dL^2^ | ***.118 ^pt^*** |
| Serum ALP: Mdn (IQR) | 268 (148 – 793) IU/L | 426 (220 – 813) IU/L | ***.004 ^wsr *^*** |
| Serum iPTH: Mdn (IQR) | 408 (101 – 952) pg/ml | 794 (280 – 1379) pg/ml | ***.011 ^wsr *^*** |

**Supplementary Table 2. Routine CKD MBD markers**

***CKD,chronic kidney disease; MBD, mineral bone disease; OL-HDF, online hemodiafiltration; x̅ ± SD, mean ± standard deviation; Mdn, median; IQR, interquartile range; cCa^2+^, corrected serum calcium; Ca x P, calcium-phosphate by product; ALP, alkaline phosphatase; iPTH, intact parathyroid hormone; ^*^, p is significant; ^wsr^ , Wilcoxon Signed Rank Test; , paired t-test***

| *Markers* | *n* | *Mdn; (IQR)* | *P value* |
| --- | --- | --- | --- |
| Basal Klotho | 23 | 4.2ng/ml; (2.3 - 5.5) | ***.168^wsr^*** |
| Klotho at 12 months | 23 | 4.4 ng/ml; (3.0 - 7.1) |  |
| Basal FGF-23 | 23 | 350.7 pg/ml (287.6 - 386.5) | ***.011 ^wsr *^*** |
| FGF-23 at 12 months | 23 | 262.2 pg/ml (154.1 – 349.6) |  |
| Basal FGF-23/Klotho | 23 | 68.1 (65.3 - 184.6) | ***.024 ^wsr *^*** |
| FGF-23/Klotho at 12 months | 23 | 62.6 (55.1 - 68.7) |  |
| Basal BALP | 23 | 104.6 IU/L; (91.1 - 111.8) | ***.004^wsr*^*** |
| BALP at 12 months | 23 | 143.2 IU/L; (91.2 – 172.9) |  |
| Basal TRAP-5b | 23 | 5.5 U/L (4.7 – 9.2) | ***.001 ^wsr *^*** |
| TRAP-5b at 12 months | 23 | 3.6 U/L (3.1– 4.8) |  |
| Basal BALP/TRAP-5b | 23 | 20.2 (10.8 – 20.8) | ***.001^wsr*^*** |
| BALP/TRAP-5b at 12 months | 23 | 31.9 (20.7 – 49.6) |  |

**Supplementary Table 3. Impact of OL-HDF on Klotho, FGF-23, BALP and TRAP-5b in the subgroup of patients having convective volume more than 12L/m^2^**

***n_,_ patients having convective volume more than 12L/m^2^; Mdn, median; IQR, interquartile range); ^*^, P value is significant; ^wsr^ , Wilcoxon Signed Rank Test***

| *Markers* | *n* | *Mdn; (IQR)* | *P value* |
| --- | --- | --- | --- |
| Basal Klotho | 18 | 3.7 ng/ml; (2.3 - 5.4) | ***.068* ^wsr^** |
| Klotho at 12 months | 18 | 5.9 ng/ml; (3.5 - 8.4) |  |
| Basal FGF-23 | 18 | 360.3 pg/ml (251.8 - 515.5) | ***.010 ^wsr *^*** |
| FGF-23 at 12 months | 18 | 257.8 pg/ml (138.9 - 345.6) |  |
| Basal FGF-23/Klotho | 18 | 68.6 (66.0 - 194.2) | ***.028 ^wsr *^*** |
| FGF-23/Klotho at 12 months | 18 | 58.6 (36.9 - 68.6) |  |
| Basal BALP | 18 | 103.0 IU/L; (91.9 - 112.0) | ***.002^wsr*^*** |
| BALP at 12 months | 18 | 151.8 IU/L; (104.7 – 173.4) |  |
| Basal TRAP-5b | 18 | 5.5 U/L (4.9 – 9.5) | ***.008 ^wsr *^*** |
| TRAP-5b at 12 months | 18 | 3.9 U/L (3.2 – 4.9) |  |
| Basal BALP/TRAP-5b | 18 | 20.3 (10.3 – 20.9) | ***.002^wsr*^*** |
| BALP/TRAP-5b at 12 months | 18 | 34.8 (21.2 – 51.0) |  |

**Supplementary Table 4. Impact of OL-HDF on Klotho, FGF-23, BALP and TRAP-5b in the subgroup of patients having convective volume more than 12L/m^2^ and were basically on hemodialysis with high flux membrane**

***n_,_ patients having convective volume more than 12L/m^2^ and dialyzed at baseline with high flux dialyzers; Mdn, median; IQR, interquartile range; ^*^, P value is significant; ^wsr^ , Wilcoxon Signed Rank Test***

|  | ***n^a^*** | ***x̅ ± SD*** | ***P value*** |
| --- | --- | --- | --- |
| **Basal dry weight** | 31 | 27.04 ± 8.03 kg | ***< .0001^a*^*** |
| **Dry weight at 12 months** | 31 | 28.27 ± 8.28 kg |  |
| **Basal dry weight z score** | 31 | -2.54 ± -1.49 | ***< .0001^a*^*** |
| **Dry weight z score at 12 months** | 31 | -2.99 ± -1.66 |  |
|  |  |  |  |
| **Basal height** | 31 | 127.09 ± 14.8 cm | ***< .0001^a*^*** |
| **Height at 12 months** | 31 | 130.51 ± 15.21cm |  |
| **Basal height z score** | 31 | -2.99 ± -1.08 | ***.130^a^*** |
| **Height z score at 12 months** | 31 | -3.09 ± -1.11 |  |
|  | ***n^b^*** |  |  |
| **Basal height** | 20 | 130.65 ± 11.56 cm | ***< .0001^a*^*** |
| **Height at 12 months** | 20 | 134.33 ± 12.55 cm |  |
| **Basal height z score** | 20 | -2.64 ± - 0.85 | .***153^a^*** |
| **Height z score at 12 months** | 20 | -2.74 ± -0.91 |  |
|  |  |  |  |

**Supplementary Table 5. Impact of OL-HDF on weight and height**

***n^a^, a ll patients; n^b^, after exclusion of patients with deformities; x̅ ± SD, mean ± standard deviation; ^a^, Paired t-Test; ^*^, P value is significant***

| Parameters | Baseline | At 12 months of OL-HDF | P value |
| --- | --- | --- | --- |
| Frequency |  |  |  |
| Alfacalcidol | 23 (74.2%) | 21 (67.7%) | ***.431*** |
| Calcium supplements | 20 (64.5) | 17 (54.8%) | ***.823*** |
| Phosphate binder (Sevelamer) | 11 (35.5%) | 10 (32.3%) | ***.691*** |
| Calcimimitics (Cinacalcet) | 5 (16.1) | 5 (16.1) | ***.172*** |
| Growth hormone | None | None |  |
| Dose |  |  |  |
| Alfacalcidol | 7.0 (0.0 – 7.0) µg/week | 7.0 (4.0 – 7.0) µg/week | ***.051*** |
| Calcium supplements | 30.0 (23.5 – 53.8) mg/Kg/day | 20.0 (0.0 – 56.0) mg/Kg/day | ***.289*** |
| Phosphate binder (Sevelamer) | 1.0 (0.0 – 2.0) tablet/day | 0.0 (0.0 – 1.0) tablet/day | ***.071*** |
| Calcimimitics (Cinacalcet) | 0.0 (0.0 – 0.0) mg/day | 0.0 (0.0 – 30.0) mg/day | ***.063*** |

**Supplementary Table 6. Impact of OL-HDF on frequency and dose of medications**

***OL-HDF, online hemodiafiltration; Data are presented as number (n) with percentage or as median and interquartile range.***
